# A clinical comparison of etomidate-lipuro, propofol and admixture at induction

**DOI:** 10.4103/1658-354X.76509

**Published:** 2011

**Authors:** Fatma Saricaoglu, Sennur Uzun, Oguzhan Arun, Funda Arun, Ulku Aypar

**Affiliations:** *Department of Anesthesiology and Reanimation, Ankara, Turkey*

**Keywords:** *Bispectral index*, *etomidate*, *propofol lipuro*, *hemodynamics*, *injection pain*, *myoclonus*

## Abstract

**Objective:**

The purpose of this study was to compare etomidate-lipuro and propofol and 50%, (1:1) admixture of these agents at induction with special reference to injection pain, hemodynamic changes, and myoclonus.

**Methods:**

Ninety patients were assigned at random to three groups in which induction was performed with either etomidate-lipuro, propofol or etomidate-lipuro–propofol admixture. After monitorization with bispectral index (BIS) all agents were given with infusion with a perfuser at a constant rate of 200 ml/min till the BIS values decreased to 40. Blood pressure and heart rate were measured every 30 s at this period. Patients were asked for pain at the injection site and observed visually for myoclonus. The time BIS values decreased to 40 (BIS 40 time) and total amounts of induction doses were measured.

**Results:**

BIS 40 time measurements were P > E > PE (199.4 ± 40.9, 176.9 ± 31.6, 163.5 ± 20.6 s). The hemodynamic (systolic, diastolic and mean blood pressures, heart rate) changes were minimal in group PE than other two groups (*P* = 0.017). The intensity of myoclonus was graded as mild in 9, moderate in 12, and severe in 5 patients in the group E (76.3%). Myoclonus was not observed in group PE and group P. There were no injection pain in group PE as the incidence were (83.8%) in group P and in (63.2%) group E.

**Conclusion:**

Incidence of hemodynamic changes, myoclonus, and injection pain is significantly lower in group PE. BIS 40 times is least in group PE. We concluded that 1:1 admixture of etomidate-lipuro and propofol is a valuable agent for induction.

## INTRODUCTION

Propofol (propofol 1% fresenius, Fresenius Kabi AB., Germany) is a nonopioid, nonbarbiturate, sedative-hypnotic agent with rapid onset and short duration of action. Adverse effects include hypotension and injection pain.[1,2]

Etomidate is a hypnotic agent causing minimal histamine release and very stable hemodynamic profile. However, pain on injection and myoclonus are the most common side effects of this drug.[3] Pains on injection, venous irritation and hemolysis have been abolished by new fat emulsion of etomidate (Medium chain triglyceride and soya bean named Etomidate – Lipuro, B.Braun, Melsungen, Germany), but the new solvent has not reduced the incidence of myoclonus after etomidate injection. Myoclonus is a serious problem in patients either with open globe injury or emergency nonfasting conditions.[4]

Bispectral index (BIS) monitoring has emerged as convenient and versatile tool to titrate hypnotic agents and to reduce drug consumption. BIS is a dimensionless number scaled from 100 to 0, with 100 representing an awake electroencephalogram and 0 representing electrical silence.[5]

The aim of the present prospective randomized controlled single centre double-blind trial was to evaluate the effect of intravenous propofol/etomidate-lipura combination (etofol) in the same syringe for induction in general anesthesia in reference to doses for same BIS values, hemodynamics, myoclonus, and injection pain.

## METHODS

Following written patient consent, 89 patients listed for various types of surgery were scheduled to be included (ASA I–II). The study used a single centre prospective, randomized, double blind controlled design. Randomization was based on computer generated random numbers. The study was approved by University Ethics Committee and adhered to Good Clinical Practise (GCP) guidelines. Patients with vascular diseases, habituation to analgesics, sedatives or antianxiety drugs; allergic diseases or sensitivity to propofol or etomidate, lipid emulsions

The syringes containing the study drugs were prepared by the same anesthesia resident to assure a proper blinding procedure. The coded syringes contained either etomidate-lipuro 2 mg/ml or propofol 1% fresenius 20 mg/ml or etofol prepeared as 1:1 mixture of propofol 1% fresenius 20 mg/ml and etomidate-lipuro 2 mg/ml. The syringes were prefilled to contain 20 ml for blinding purposes (no visual difference could be detected between syringes).

Upon arrival in the operating room, patients were equipped with a Standard anesthesia monitoring (Datex-Ohmeda ™ s/5 ™, Helsinki, Finland). The BIS was monitored using the XP device (version 4.0) and specific quatro sensor (Aspect Medical Systems, Newton, MA, USA and Leiden, The Netherlands). The BIS sensor was appropriately applied on the left side of the forehead. In all patients general anesthesia was induced using a perfuser at a constant rate 200ml h with all three agents. During induction, three agents were titrated until the target level of BIS 40 was obtained; the patients were ventilated by face mask with 100% O_2_. As soon as BIS was decreased to index values of 40, the investigation was completed and additional propofol, opioid, and muscle relaxant were given and anesthesia was continued according to standard clinical practice.

### Assessments

Systolic, diastolic and mean blood pressure, heart rates were recorded at every 30 s until the BIS 40 values.

Injection pain was measured using four-graded scale (0: no pain, 1: verbal complain of pain, 2: withdrawal of the arm, 3: both verbal complain and withdrawal of the arm) as described previous study.[6] Pain was assessed by the same resident (OA) in all patients. In order to preserve blinding the score was noted immediately after the patient lost consciousness, thus before possible myoclonic activity would potentially occur.

Patients were observed visually for myoclonus, and when present, myoclonus severity was graded by a trained blind resident (OA). The degree of such muscular activity was scored as follows: 0 = no myoclonus; 1 = minor myoclonus; 2 = moderate myoclonus; 3 = severe myoclonus.[3]

### Statistical analysis

Data are presented as the mean (S.D.). Categorical data are described as number of patients (*n*). Physical characteristics, SBP, MBP, and BIS values all time intervals were compared using a one-way ANOVA after normal distribution had been ascertained by Kolmogorov– Simirnov test. All categorical data including the incidence of myoclonus were compared using the χ^2^ -test. The *post hoc* Bonferroni correction was used to compare the two groups. All differences were considered significant at *P* < 0.05. Statistical analysis was performed using SPSS 13.0.

## RESULTS

Demographic data were similar between three study groups [Table 1].

**Table 1:** Culture results of ocular specimens

	Group P	Group E	Group etofol
Age (year)	34 (28–41)	36 (31–42)	41 (35–47)
Weight (kg)	68 (62–74)	71 (66–75)	76 (72–80)
Height (cm)	169 (158–178)	171 (156–180)	165 (155–178)
Gender (M/F)	11/16	14/16	17/15
Dosage (mL)	11.4 (±1.9)	9.8 (±1.8)	95 (±15)
Dosage (mg) (mean ±SD)	114(±19)	19.6 (±3.6)	47.5 P+8.50E

*P*<0,000; Results of statistical analysis are also provided as (mean [min-max])

**Figure 1:**
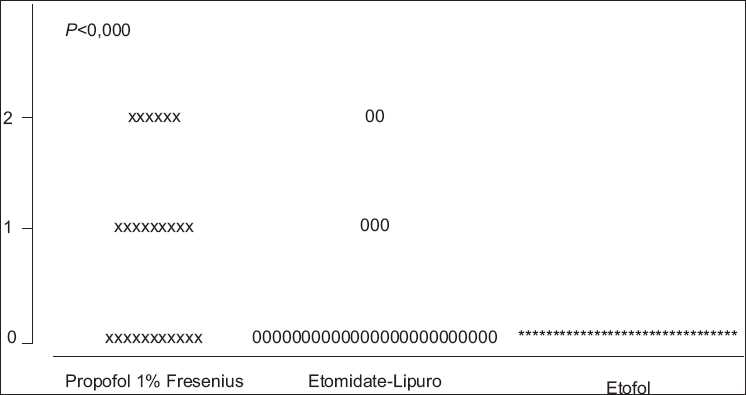
Incidence of injection pain, etofol vs etomidate-lipuro and propofol 1% fresenius

The incidence of injection pain was significantly lower in Etofol group. There were no injection pain in group PE as the incidence were (83.8%) in group P and in (63.2%) group E. The distribution of pain scores is shown in Figure 1.

**Figure 2:**
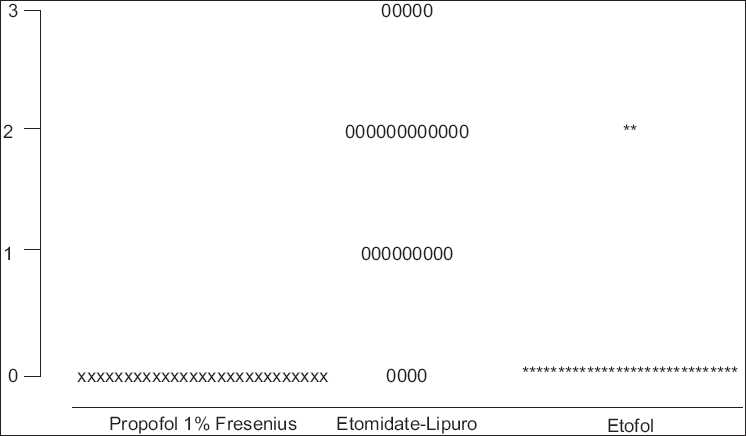
Incidence of myoclonus, etofol vs etomidate-lipuro and propofol 1% fresenius

A higher incidence of myoclonic activity was seen in etomidate-lipuro group (93.4%) compared with propofol fresenius and etofol groups (0% and 14.3%, respectively) (*P* < 0.000). The distribution of myoclonic movement scores is shown in Figure 2.

**Figure 3:**
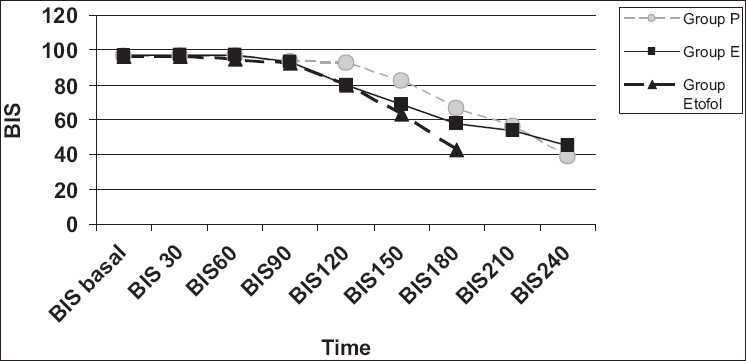
Time to reach BIS to 40 (Induction times of the groups).

The dosage for induction was significantly higher in propofol group than etomidate and etofol group in milliliter range (*P* < 0.000) [Table 1]. Induction (time to reach BIS to 40) was faster in etofol group (163.5(±20.6) s) than propofol (119.4(±40.9) s) (*P* < 0.000) and etomidate group (176(±31.6)s (*sP* = 0.026) [Figure 3].

**Figure 4:**
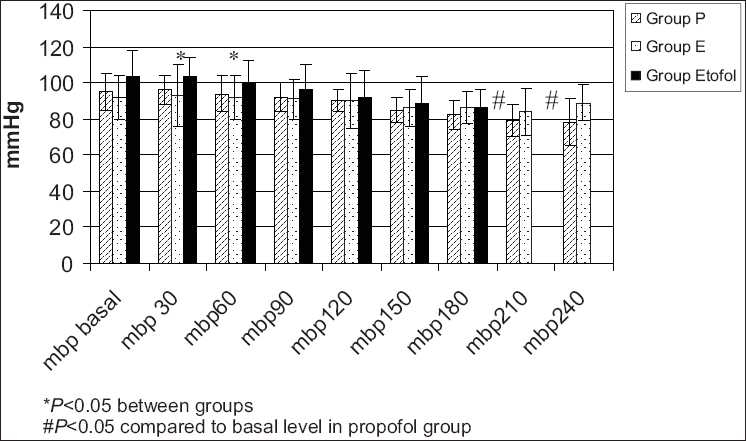
The mean blood pressures were significantly decreased in propofol group compared to etomidate and etofol group at 30 and 60 seconds and significantly lower than the basal levels at 210 and 240 seconds (The mean blood pressure measurements of the groups).

**Figure 5:**
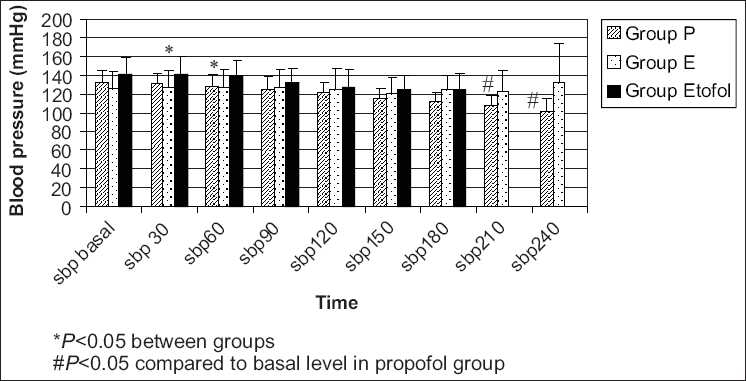
The systolic blood pressures were significantly decreased in propofol group compared to etomidate and etofol group at 30 and 60 seconds and significantly lower than the basal levels at 210 and 240 seconds

The mean and systolic blood pressures were significantly decreased in propofol group compared to etomidate and etofol group at 30 and 60 s and significantly lower than the basal levels at 210 and 240 [Figures 4 and 5].

## DISCUSSION

The main finding of the present study was the use of etomidat-lipuro-propofol 1:1 admixture for induction of anesthesia associated with no injection pain, a very low rate of myoclonus and significantly faster induction time compared to propofol and etomidate lipuro with hemodynamic stability.

In this study, we want to examine the of etomidat-lipuro-propofol 1:1 admixture for induction of anesthesia and compare the problems that can be occurred when we use these agents separately like injection pain and myoclonus.

A serious problem with the use of propofol is the high incidence of pain on injection. The currently most common practice to reduce this problem is by adding lidocaine to the propofol solution but despite this the incidence of pain on injection remains unacceptably high (20%–39%).[7,8]

Etomidate is a well-known substituted imidazole induction agent that is associated with very high degree of hemodynamic stability.[4] A problem with etomidate was that the solvent used also caused pain on injection. The side effects of etomidate dissolved in PG can be eliminated while retaining the profile of actions when etomidate is dissolved in lipofundin (medium chain triglycerides).[6] This drug preparation has now been approved and is available in numerous European and non-European countries (etomidate lipuro, B. Braun Melsungen, Melsungen, Germany). Etomidate dissolved in lipofundin has an almost physiological osmolality and is devoid of pain upon injection, postoperative thrombophlebitis or hemolytic effects.[6,9]

These two drugs were studied at Hacettepe University, Faculty of Pharmacy (Department of Pharmaceutical Technology) for availability for admixture. They reported that these dugs can be mixed and physically available for an admixture; we named this admixture etofol.

The use of etofol was found to significantly reduce the incidence of pain on injection, compared with propofol and etomidate-lipuro. It is already reported that etomidate-lipuro is associated with significantly less pain on injection than propofol added lidocain in children.[6] In our study no incidence of pain on injection was observed in etofol group but the incidence was significantly high in propofol group than etomidate lipuro group.

The decrease in pain when using LCT/MCT in etomidate lipuro is considered to be attributed to the lipid solvent that decreases the propofol concentration in the Etofol group.[9,10] Thus, it is possible that a reduction in either bradykinin generation or the propofol concentration decreases the pain on injection with propofol.[11]

The other disturbing side effect is myoclonus especially with etomidate. Fifty to eighty percent of unpremedicated patients may develop myoclonic movements after etomidate administration.[12,13] Myoclonus is especially problematic in nonfasting patients, patients with open eye injuries, or those who have limited cardiovascular reserves. A myoclonus from pain response on injection of etomidate was distinguished by timing of the assessments. We assessed injection pain while we were infusing the etomidate, but myoclonus was assessed after etomidate in order to differentiate among different effects of the pretreatment drugs on pain and on myoclonus.

In agreement with previous literature the use of etomidate was found to be associated with higher incidence of myoclonic activity than propofol. There were only two patients at stage 2 in etofol group where as no patient with myoclonus in propofol group. There was not any statically significance among myoclonus between propofol and etofol group (*P* > 0.05). Previous studies in adults have also shown that the incidence of myoclonic movements can be reduced either by premedication with fentanyl or by preinduction priming with subanesthetic dose etomidate.[12] The propofol in the mixture can be act as preinduction priming in our study.

The induction time (Time to reach BIS to 40) is faster in Etofol group than propofol and etomidate group (etofol > propofol > etomidate). The rapid induction without any side effect is a valuable characteristic that wanted from an ideal induction agent. Both etomidate and propofol are known to have short duration of action that will allow rapid induction.

Hemodynamic stability is the other part of the ideal induction agent. Although propofol decreased the blood pressure Etofol is associated with hemodynamic stability as etomidate in our study.

We did not examine the cortisol levels of the groups. Although etomidate causes adrenocortical suppression, a single injection to induce anesthesia will only produce a transient and clinically insignificant interference with adrenocortical function.[14] In a study by Schenarts and colleagues the use of etomidate for induction of anesthesia in emergency department, as compared with midazolam, was associated with reduced cosyntropin stimulation test response (30% of the control group response) at 3 h after administration.[15] However, cosyntropin stimulation test response was back to normal at 12 h after the administration. Furthermore the reduce adrenal response to cosyntropin during the early phase after administration, the serum cortisol levels remained within the normal laboratory reference ranges during limited period of adrenal inhibition.

In conclusion, etofol (1:1 admixture of etomidate-lipuro and propofol) is associated with less pain on injection, myoclonus and with hemodynamic stability than etomidate lipuro and propofol and we think it is a valuable agent for induction.
